# Predictors of adherence to wearing therapeutic footwear among people with diabetes

**DOI:** 10.1186/s13047-020-00413-z

**Published:** 2020-07-13

**Authors:** Gustav Jarl, Roy Tranberg, Ulf Johansson, John Alnemo, Lars-Olov Lundqvist

**Affiliations:** 1 Department of Prosthetics and Orthotics, Faculty of Medicine and Health, Örebro University, Örebro, Sweden; 2 University Health Care Research Center, Faculty of Medicine and Health, Örebro University, Örebro, Sweden; 3 Department of Orthopaedics, Institute of Clinical Sciences, the Sahlgrenska Academy at University of Gothenburg, Gothenburg, Sweden; 4 Department of Prosthetics and Orthotics, Sahlgrenska University Hospital, Gothenburg, Sweden

**Keywords:** Diabetic foot, Foot ulcer, Treatment adherence and compliance, Patient compliance, Shoes

## Abstract

**Aims:**

People at increased risk of developing diabetic foot ulcers often wear therapeutic footwear less frequently than is desirable. The aims were to identify patient groups prone to nonadherence to wearing therapeutic footwear and modifiable factors associated with adherence.

**Materials and methods:**

A questionnaire was mailed to 1230 people with diabetes who had been fitted with therapeutic footwear. Independent variables were categorized into five domains. For each domain, variables that were associated with adherence in a univariate regression analysis were entered into a multiple regression analysis.

**Results:**

A total of 429 (34.9%) questionnaires were analyzed. Multiple regression analyses showed significant associations (*p* < 0.05) between higher adherence and paid employment, current foot ulcer, previous foot ulcer, satisfaction with follow-up, self-efficacy, understanding of lost/reduced sensation as a risk factor for foot ulcerations, visible storage of therapeutic footwear at home, storage of conventional footwear out of sight, consistent choices about which footwear type to wear, and a belief that therapeutic footwear promotes ulcer healing. The five multivariate models explained 2–28% of the variance in adherence, with the strategies for footwear use domain explaining the most.

**Conclusions:**

Patients without paid employment or without foot ulcer experience are more prone to nonadherence. To improve adherence, clinicians should advise patients to store therapeutic footwear in a visible place at home and put conventional footwear away and encourage patients’ self-efficacy and habitual use of therapeutic footwear. Future studies should investigate this topic further and explore ways to promote changes in habits. A study limitation was that all variables were self-reported.

## Introduction

Diabetic foot ulcers affect 19–34% of people with diabetes during their lifetimes and are associated with increased mortality and risk of amputation [1]. Although most ulcers heal, recurrence rates are alarmingly high: approximately 40% of patients develop a new ulcer within 1 year after healing, and this figure increases to 60 and 65% after 3 and 5 years, respectively [1]. Evidence-based guidelines and systematic reviews recommend that people with previous plantar foot ulcers wear therapeutic footwear to prevent reulceration [2, 3], but the level of adherence to wearing therapeutic footwear is often lower than desirable [4–7]. A review from 2016 [8] identified only six quantitative observational studies that investigated footwear adherence and did not find strong evidence for the ability of any factor to predict adherence. Two studies in the review found that perceiving the benefits of wearing therapeutic footwear was associated with a higher degree of adherence [4, 6]. Several other factors were associated with adherence in some studies but not in others (age [4, 6, 7, 9], body mass index [4, 7], diabetes type [4, 7, 9], foot deformity and minor amputation [4, 7, 9], perceived severity of the foot condition [6, 9], and therapeutic footwear appearance [4, 7, 9]). After searching in the relevant databases for more recent publications, we found only one quantitative observational study. In that study, adherence was not significantly different between men and women [10]. Interventional studies to improve adherence are even more rare. We are aware of only one small experimental study, which found that motivational interviewing improved adherence in the short term but that adherence returned to the low baseline level in the long term [11]. Thus, it is still uncertain which patient groups are in most need of interventions to improve adherence and what variables should be the targets for such interventions. The aims of the study were to identify patient groups prone to nonadherence to wearing therapeutic footwear and modifiable factors associated with adherence.

## Materials and methods

A questionnaire was constructed based on the Health Belief Model, which includes perceived seriousness of the health condition, perceived susceptibility of developing the condition, perceived benefits and barriers to engaging in the health behavior, cues to action, and self-efficacy [12]. The model predicts that people who have better adherence to wearing therapeutic footwear have the following perceptions: foot ulcers are serious and they are susceptible to developing diabetic foot ulcers, there are substantial benefits and few obstacles to wearing therapeutic footwear, and they have a high degree of self-efficacy and experience some cue prompting them to wear the therapeutic footwear. The model has previously been used to study diabetic foot self-care adherence [13] but not to study adherence to wearing therapeutic footwear. The questionnaire included items covering demographics, diabetes type, foot complications, satisfaction with services, understanding of sensory neuropathy as a risk factor for foot ulcers, internal locus of control, belief in the efficacy of therapeutic footwear, concerns about the prevention and healing of foot ulcers, self-efficacy, general health, depression, attitudes towards therapeutic and conventional footwear, footwear adherence, reminders to use therapeutic footwear, and social support. Some items were constructed for this study, while the majority of the items were copied or adapted from existing validated instruments [14–22]. The questionnaire was pilot tested on five people with experience with diabetic foot ulcers [5]. They answered the questionnaire and were subsequently interviewed individually on their understanding of the content. After minor revisions, the questionnaire was mailed to all people (*n* = 1230) fulfilling the criteria for inclusion in the study in May and June 2017. The inclusion criteria were that the person should be at least 18 years old (on January 1, 2016), have diabetes mellitus, have been prescribed therapeutic footwear at some point and have visited one of the two participating prosthetic and orthotic clinics from January–December 2016. Bilateral major amputation was the only exclusion criterion. For those who had not responded to the questionnaire 1 month later, a reminder letter was sent. The study was approved by the Regional Ethics Committee Review Board of Uppsala (reference number 2016/528).

### Statistical methods

The respondents’ age and sex distributions were compared to those of the rest of the sample, using a two-sided t-test and χ^2^-test, respectively, with *p* < 0.05 indicating a statistically significant difference. The dependent variable, adherence to wearing therapeutic footwear, was measured with two questions adapted from the Questionnaire for Persons with a Transfemoral Amputation [16]. The questions asked about the time of wearing therapeutic footwear in terms of both number of days per week (scale: 0 to 7) and number of hours per day (scale: 0–3, 4–6, 7–9, 10–12, 13–15, and more than 15). An index was calculated by multiplying the number of days/week by the number of hours/day and dividing this value by 108.5 (the number of waking hours per week, assuming 15.5 waking hours per day). For example, for a person wearing therapeutic footwear 7 days per week and 10–12 h per day, the index would be 7*11/108.5 = 0.71, indicating that the person wears therapeutic footwear 71% of the waking day.

The independent variables were grouped into five domains. The first domain consisted of variables related to demographics, health and social support with the aim of identifying patient groups prone to nonadherence. The second to fifth domains consisted of variables related to health care services, attitudes towards foot ulcers, strategies for footwear use and attitudes towards footwear. For the variables in these domains, the aim was to identify modifiable factors that were associated with adherence. Linear regression was used to test the associations between each variable and therapeutic footwear adherence. For each domain, variables with a *p*-value < 0.10 were entered into a forward linear multiple regression analysis, in which *p*-values < 0.05 were considered statistically significant. In addition, explorative secondary analyses were conducted on variables in the domain that explained most of the variance in adherence. In these analyses, adherence levels were compared between response categories for each item using one-way analysis of variance (ANOVA) with Fisher’s least significant difference (LSD) test as a post hoc test. *P*-values < 0.05 were considered statistically significant. IBM SPSS Statistics for Windows, version 25.0 (Armonk, NY: IBM Corp.) was used for all statistical analyses.

## Results

In total, 469 valid questionnaires were returned, but 26 were excluded because the respondents stated that they did not own therapeutic footwear, and 14 were excluded because they had missing answers to the questions on therapeutic footwear adherence (dependent variable). Thus, 429 questionnaires were included in the analysis, for a response rate of 34.9%. The sex distributions of the respondents (*n* = 429) and the rest of the sample (*n* = 801) were not significantly different (66.7% men vs 62.1% men, *p* = 0.112). Mean age also did not significantly differ between respondents (mean 69.1 years, standard deviation 10.6) and the rest of the sample (mean 69.6 years, standard deviation 13.3; *p* = 0.519). The majority of the respondents were men, retired, had type 2 diabetes, were ulcer-free at the time of the survey and had a history of foot ulcers (Table 1). A minority of the respondents had amputations. On average, the respondents wore their therapeutic footwear 50.3% of the waking day (standard deviation 32.8%).

**Table 1:** Demographics, health and social support in relation to adherence (*n* = 429)

				Univariate regression		Multiple regression	
Variables		N (%)	Adherence, mean (SD)	β	***p***-value	β	***p***-value
Sex							
Men		287 (66.9)	0.49 (0.32)	.041	.395		
Women		142 (33.1)	0.52 (0.35)				
Age, mean (SD)		69.1 (10.6)	0.50 (0.33)	−.052	.285		
Education							
Incomplete elementary schooling		11 (2.7)	0.48 (0.34)	−.010	.841		
Elementary school		143 (34.8)	0.49 (0.34)	−.014	.780		
Upper secondary school		152 (37.0)	0.51 (0.35)	.023	.638		
College/university		105 (25.5)	0.50 (0.29)	−.007	.889		
Occupation†							
Retired ‡		296 (70.1)	0.48 (0.33)	−.088	.072	−.004	.946
Paid employment ‡		71 (16.8)	0.61 (0.31)	.146	.003	.154	.002*
Unemployed		11 (2.6)	0.47 (0.33)	−.014	.769		
Student		3 (0.7)	0.52 (0.20)	.004	.932		
Disability pension		57 (13.5)	0.53 (0.34)	.041	.401		
Sick leave		25 (5.9)	0.56 (0.32)	.049	.312		
Diabetes type							
Type 1 ‡		118 (27.5)	0.55 (0.34)	.087	.070	.071	.151
Type 2 ‡		309 (72.0)	0.49 (0.33)	−.081	.093	−.065	.192
Other type		2 (0.5)	0.32 (0)	.038	.436		
Current foot ulcer							
Yes ‡		131 (30.8)	0.58 (0.32)	.150	.002	.108	.032*
No		295 (69.2)	0.47 (0.33)				
Previous foot ulcer							
Yes ‡		250 (60.1)	0.54 (0.33)	.158	.001	.131	.009*
No		166 (39.9)	0.44 (0.32)				
Partial foot amputation							
Yes		62 (14.8)	0.60 (0.32)	.122	.012	.063	.222
No		356 (85.2)	0.48 (0.33)				
Amputation through or above the ankle							
Yes ‡		19 (4.5)	0.65 (0.32)	.096	.049	.069	.169
No		401 (95.5)	0.49 (0.33)				
Is there someone close to you who supports you with your foot problems?							
Yes		258 (61.7)	0.50 (0.33)	.004	.940		
No		160 (38.3)	0.50 (0.33)				
		**Mean (SD**)					
Feeling down, depressed, or hopeless (1 = Almost every day to 4 = Not at all)		1.8 (1.0)	0.51 (0.33)	−.013	.798		
General health (1 = Excellent to 5 = Poor)		2.6 (1.0)	0.50 (0.33)	.038	.432		

*SD* Standard deviation. †The percentages add up to more than 100% because more than one alternative could be chosen. ‡ Variables with *p*-values < 0.10 in the univariate analyses were entered in the multiple regression analysis by domain. * *p* < 0.05 in the multiple regression analysis. Multiple regression model F_(3,394)_ = 8.04, *p* < 0.001, *R*^*2*^ = 0.06

### Domain 1. Demographics, health and social support

In the univariate regression analyses, being retired, having paid employment, having type 1 diabetes, having type 2 diabetes, having a current foot ulcer, having a previous foot ulcer, having a partial foot amputation, and having an amputation through or above the ankle had *p*-values less than 0.10 (Table 1). In the multiple regression analysis, having paid employment, having current foot ulcers and having previous foot ulcers were significant (*p* < 0.05). The model explained 6% of the variance in adherence.

### Domain 2. Health care services

All three items in this domain (responsiveness of clinic staff, partnership of the patient in clinical decision making, and satisfaction with the follow-up of footwear) had *p*-values less than 0.10 (Table 2). Only satisfaction with follow-up was significant in the multiple regression analysis, which explained 5% of the variance in adherence.

**Table 2:** Health care services, attitudes towards foot ulcers and strategies for footwear use in relation to adherence

		Univariate regression		Multiple regression	
Variables	Mean (SD)	β	*p*-value	β	*p*-value
**Health care services**					
The staff were responsive to my concerns and questions (1 = Disagree to 3 = Strongly agree) †	2.5 (0.6)	.111	.029	.017	.767
I was a partner in decision making with clinic staff (1 = Disagree to 3 = Strongly agree) †	2.4 (0.6)	.084	.098	−.018	.743
I am satisfied with the follow-up of my therapeutic footwear (1 = Not satisfied at all to 5 = Very satisfied) †	3.8 (1.2)	.236	<.001	.226	<.001*
**Attitudes towards foot ulcers**					
Lost/reduced sensation in your feet increases the risk of foot ulcerations (1 = Strongly disagree to 5 = Strongly agree) †	3.9 (1.2)	.157	.001	.153	.002*
What you do yourself is the main thing that affects whether you develop new foot ulcers (1 = Strongly disagree to 5 = Strongly agree) †	3.7 (1.2)	.032	.519		
Worried about getting new foot ulcers in the future (1 = Not at all to 5 = Very much) †	3.0 (1.4)	.099	.044	.051	.327
**Strategies for footwear use**					
Confident I would always wear therapeutic footwear if I decided to do so (1 = Very uncertain to 4 = Very certain) †	3.1 (1.0)	.403	<.001	.201	<.001*
	**n (%)**				
Where do you keep your therapeutic footwear? †		−.241	<.001	−.171	.001*
Put away, e.g., in a wardrobe	26 (6.3)				
Visible at home	384 (93.7)				
Where do you keep your conventional footwear? †		.330	<.001	−.159	.004*
Visible at home	198 (47.1)				
Put away, e.g., in a wardrobe	135 (32.1)				
Do not own conventional footwear	87 (20.7)				
How do you choose between wearing therapeutic and conventional footwear? †		.383	<.001	.271	<.001*
Decide each time	141 (42.6)				
I always do it the same way	190 (57.4)				
Does someone usually remind you to wear your therapeutic footwear? †		−.099	.043	.689	.994
Yes, clinic staff and/or people close to me	95 (22.9)				
No	320 (77.1)				

*SD* Standard deviation. † Variables with *p*-values < 0.10 in the univariate analyses were entered in the multiple regression analysis per domain. * *p* < 0.05 in the multiple regression analysis

Multiple regression model for the Health care services domain, F_(1,374)_ = 20.10, *p* < 0.001, *R*^*2*^ = 0.05; Attitudes towards foot ulcers domain, F_(1,400)_ = 9.58, *p* < 0.002, *R*^*2*^ = 0.02; and Strategies for footwear use domain, F_(4,309)_ = 28.98, *p* < 0.001, *R*^*2*^ = 0.28

### Domain 3. Attitudes towards foot ulcers

Understanding that lost or reduced sensation in the feet increases foot ulceration risk and being worried about developing new foot ulcers had *p*-values less than 0.10 in the univariate regression analyses, but only the former was significant (*p* < 0.05) in the multiple regression analysis (Table 2). The model explained 2% of the variance.

### Domain 4. Strategies for footwear use

All variables in this domain had *p*-values less than 0.10 in the univariate regression analyses (Table 2). In the multiple regression analysis, four variables were significantly associated with adherence: self-efficacy (being confident about being able to wear therapeutic footwear all the time), storage of therapeutic footwear, storage of conventional footwear and approach to making choices about what footwear type to wear. The model explained 28% of the variance in adherence.

### Domain 5. Attitudes towards footwear

All but one variable in this domain had *p*-values less than 0.10 in the univariate regression analyses (Table 3). Only perception of footwear’s effect on ulcer healing was significant in the multiple regression analysis, which explained 11% of the variance in adherence.

**Table 3:** Attitudes towards footwear in relation to adherence

Variables	Mean (SD)	Univariate regression		Multiple regression	
		β	***p***-value	β	***p***-value
1. Effect on ulcer healing †	4.2 (0.8)	.367	<.001	.324	<.001*
2. Effect on reducing the risk of new ulcers †	4.2 (0.8)	.312	<.001	.127	.137
3. Difficulties walking in the footwear †	3.7 (1.1)	.308	<.001	.103	.110
4. Appearance †	2.6 (1.2)	.102	.043	−.014	.815
5. Weight †	2.7 (1.1)	.168	.001	.070	.242
6. Price †	3.4 (1.2)	.145	.006	.038	.531
7. Pain when standing and walking †	3.9 (1.0)	.277	<.001	.101	.138
8. Difficulties putting on and taking off the footwear †	3.4 (1.0)	.155	.002	.055	.361
9. Ease of use in everyday activities, e.g., in your work †	3.7 (1.1)	.246	<.001	.094	.140
10. Feeling inclined to wear the footwear in public †	3.0 (1.1)	.107	.034	−.053	.380
11. Fit of the footwear †	3.8 (1.1)	.274	<.001	.079	.203
12. Probability of new ulceration within 12 months if I always wear therapeutic footwear	2.0 (0.8)	.026	.609		
13. Probability of new ulceration within 12 months if I never wear therapeutic footwear †	2.8 (1.0)	.182	<.001	.078	.197

*SD* standard deviation. Response options: for items 1–11, 1 = Conventional footwear is much better to 5 = Therapeutic footwear is much better, where 3 indicates indifference; for items 12–13, 1 = Highly improbable to 4 = Highly probable. † Variables with *p*-values < 0.10 in the univariate analyses were entered in the multiple regression analysis. * *p* < 0.05 in the multiple regression analysis

Multiple regression model, F_(1,261)_ = 28.02, *p* < 0.001, *R*^*2*^ = 0.11

### Secondary analyses

Secondary analyses were performed on the four items that were significant in the multiple regression analysis of the Strategies for footwear use domain, as this domain explained the largest amount of variance (Table 4). In a comparison of the highest and lowest response categories for each item, the mean adherence differed between 0.25 and 0.35 across items. When the two items about footwear storage were combined for people who owned both therapeutic and conventional footwear, adherence was highest (0.61) among people who kept their therapeutic footwear visible at home and put their conventional footwear away. This value was more than six times higher than that for people with the lowest adherence (0.09), that is, people who kept their conventional footwear visible at home and put their therapeutic footwear away.

**Table 4:** Secondary analyses of selected items from the Strategies for footwear use domain

Variables	N (%)	Adherence, mean (SD)	Comparisons of adherence †
Confident I would always wear therapeutic footwear if I decided to do so			F_(3,405)_ = 28.280 (*p* < .001)
Very uncertain	46 (11.2)	0.29 (0.31)	a
Moderately uncertain	53 (13.0)	0.33 (0.29)	a
Moderately certain	125 (30.6)	0.45 (0.28)	b
Very certain	185 (45.2)	0.64 (0.31)	c
How do you choose between wearing therapeutic and conventional footwear?			F_(1,329)_ = 56.411(*p* < .001)
Decide each time	141 (42.6)	0.32 (0.28)	a
I always do it the same way	190 (57.4)	0.57 (0.30)	b
Where do you keep your therapeutic footwear?			F_(1,408)_ = 25.257(*p* < .001)
Put away, e.g., in a wardrobe	26 (6.3)	0.21 (0.26)	a
Visible at home	384 (93.7)	0.53 (0.32)	b
Where do you keep your conventional footwear?			F_(2,417)_ = 28.080 (*p* < .001)
Visible at home	198 (47.1)	0.39 (0.30)	a
Put away, e.g., in a wardrobe	135 (32.1)	0.59 (0.32)	b
Do not own conventional footwear	87 (20.7)	0.65 (0.31)	b
Combination of footwear storage variables			F_(3,320)_ = 19.454(*p* < .001)
Conventional footwear visible at home, therapeutic footwear put away	14 (4.3)	0.09 (0.13)	a
Therapeutic and conventional footwear put away	10 (3.1)	0.37 (0.32)	b
Therapeutic and conventional footwear visible at home	178 (54.9)	0.41 (0.30)	b
Therapeutic footwear visible at home, conventional footwear put away	122 (37.7)	0.61 (0.30)	c

*SD* Standard deviation. † When the one-way analysis of variance was significant *(p* < 0.05), pairwise post hoc comparisons were conducted using Fisher’s least significant difference (LSD) test. Different letters (a, b and c) denote that adherence was significantly different (*p* < 0.05), and the same letters denote that adherence was not significantly different

## Discussion

The first aim of the study was to identify patient groups who were prone to nonadherence. People with paid employment had higher adherence than those without paid employment, which may be because employed people spend more time away from home, where adherence is often higher than at home [7]. Furthermore, the level of adherence was higher among people with current or previous foot ulcers, suggesting that active foot complications may serve as a wake-up call to patients [23] and that additional clinical attention should be paid to people without personal experience of foot ulcers. However, differences in adherence were small between those with and without paid employment and between those with and without ulcer experience (Table 1). This may also explain why previous studies with smaller sample sizes have not found employment [7, 9] or previous foot ulcer experiences [7, 9, 24] to be significantly associated with adherence. Previous amputations were associated with adherence in the univariate analysis but not in the multiple regression analysis. This result may be explained by the fact that most amputations are preceded by a foot ulcer [25], and thus, the ulcer and amputation variables may have overlapped. The majority of the respondents in this study were men, which is consistent with several studies that have found men to be at higher risk than women for developing foot ulcers [26]. Sex, age, education level and diabetes type were not associated with adherence. This finding is consistent with most previous research [4, 6, 7, 9, 24, 27] and suggests that basic demographic and diabetes-related characteristics are not useful for identifying nonadherent patient groups.

The second aim was to identify modifiable factors that are associated with adherence to wearing therapeutic footwear. The greatest proportion of variance was explained by the Strategies for footwear use domain. This domain explained 28% of the variance, which is more than the variance explained in a previous study on footwear adherence [7]. The secondary analyses demonstrated substantial differences in adherence between people who stored their footwear in different ways, between people with high and low self-efficacy, and between people who did or did not make consistent choices about footwear. Although these aspects have not previously been investigated in quantitative studies, the results are consistent with qualitative studies that have found that the formation of new habits is important for adherence to diabetic foot self-care and footwear use [28, 29]. The final regression model consisted of four variables (self-efficacy, conventional footwear storage, therapeutic footwear storage and habitual footwear choice) that explained a moderate proportion of the variance in adherence. This finding supports the notion that adherence is a multifactorial phenomenon [8, 28, 30]. It also indicates that the observed variables are of importance but that there still is a substantial amount of unexplained variance in adherence to therapeutic footwear. This unexplained variance may be due to other independent variables that were not measured in the present study as well as to errors in the measurement of adherence. Thus, future research should investigate additional variables, such as body mass index [7] and patients’ acceptance of their disease and need of therapeutic footwear [31], as well as use objective measures of adherence, such as temperature and activity monitors [32].

Studies on footwear adherence have been criticized for not defining the conceptual framework, resulting in high heterogeneity, and for focusing on a narrow range of factors, typically those related to the patient, therapy and health condition [32]. This study included a wide range of factors and is the first study to use the entirety of the Health Belief Model to study the predictors of footwear adherence. However, perceived benefits of therapeutic footwear, self-efficacy and cues to action were the only factors from the model that were significantly associated with adherence in the multiple regression analysis. This would suggest that the model may need to be revised or replaced by another model to better understand footwear adherence.

Although the conclusions are preliminary, the study has some clinical implications. First, patients should be advised to store their conventional footwear out of sight to eliminate the visual cue (temptation) to wear it and increase the effort to choose it by needing to go and get it from somewhere else. Second, patients should be advised to keep their therapeutic footwear visible at home to provide a visual cue (reminder) to wear it and reduce the effort to choose it. Third, clinicians should discuss with patients how to form new footwear habits, that is, how therapeutic footwear can become the new default option that is chosen without conscious effort. Strategies to create such new habits is an important avenue for future research and may include advice on how to store footwear and patient education to support self-efficacy [33], which was the factor that most strongly correlated with adherence in this study. Other suggestions for clinicians to improve adherence are to follow-up therapeutic footwear prescription and educate patients on how peripheral neuropathy increases the risk of ulcerations. Additionally, patients should be educated with the aim of strengthening their belief in the efficacy of therapeutic footwear in healing and preventing ulcers. For instance, education could include a visualization of plantar pressure measurements to compare the amounts of pressure when one wears therapeutic footwear, wears conventional footwear and walks barefoot [34]. Finally, clinicians prescribing therapeutic footwear should acknowledge that improving certain footwear attributes (e.g., fit, pain and walking difficulties) may improve adherence. However, these suggestions are preliminary, and future research is needed to explore this further.

This is the largest study on therapeutic footwear adherence to date, and the results revealed that adherence was explained to the largest extent by variables that have not previously been investigated. Thus, a strength of the study was that a wide range of potential predictors were included. Some limitations of the study were that it was observational and cross-sectional, which limited the possibility of inferring causality. For example, certain ways to store footwear, high self-efficacy and the habitual use of therapeutic footwear may improve adherence, but it is possible that these variables reflect adherence rather than cause it. Thus, future studies should use interventions to modify these variables to investigate whether they actually are causes of adherence and potential targets for clinical interventions to improve adherence. In addition, studies should be conducted in other countries to test the generalizability of the results. Other limitations were that we only had data on sex and age for the non-respondents, which means that we cannot know if the sample was representative of the full population. Furthermore, all data were self-reported, and the questionnaire’s content validity and psychometric properties were not investigated.

## Conclusions

Patients without paid employment or without experience of foot ulcers are more prone to nonadherence than those with employment or with foot ulcer experience. To improve adherence, clinicians should advise patients to store therapeutic footwear in a visible place at home and put conventional footwear away and encourage patients’ self-efficacy and habitual use of therapeutic footwear.

## Data Availability

The data cannot be shared due to patient confidentiality and Swedish law.

## Abbreviations

ANOVA: One-way analysis of variance
LSD test: Fisher’s least significant difference test

## References

[1] Armstrong DG, Boulton AJM, Bus SA (2017). Diabetic foot ulcers and their recurrence. N Engl J Med.

[2] Bus SA, Lavery LA, Monteiro-Soares M, Rasmussen A, Raspovic A, Sacco ICN, van Netten JJ, on behalf of the International Working Group on the Diabetic Foot (IWGDF) (2020). Guidelines on the prevention of foot ulcers in persons with diabetes (IWGDF 2019 update). Diabetes Metab Res Rev.

[3] van Netten JJ, Raspovic A, Lavery LA, Monteiro-Soares M, Rasmussen A, Sacco I, Bus SA (2020). Prevention of foot ulcers in the at-risk patient with diabetes: a systematic review. Diabetes Metab Res Rev.

[4] Arts ML, de Haart M, Bus SA, Bakker JP, Hacking HG, Nollet F (2014). Perceived usability and use of custom-made footwear in diabetic patients at high risk for foot ulceration. J Rehabil Med.

[5] Jarl G, Alnemo J, Tranberg R, Lundqvist L-O (2019). Gender differences in attitudes and attributes of people using therapeutic shoes for diabetic foot complications. J Foot Ankle Res.

[6] Macfarlane DJ, Jensen JL (2003). Factors in diabetic footwear compliance. J Am Podiatr Med Assoc.

[7] Waaijman R, Keukenkamp R, de Haart M, Polomski WP, Nollet F, Bus SA (2013). Adherence to wearing prescription custom-made footwear in patients with diabetes at high risk for plantar foot ulceration. Diabetes Care.

[8] Jarl G, Lundqvist L-O (2016). Adherence to wearing therapeutic shoes among people with diabetes: a systematic review and reflections. Patient Prefer Adherence.

[9] Breuer U (1994). Diabetic patient's compliance with bespoke footwear after healing of neuropathic foot ulcers. Diabet Metab.

[10] Ehrmann D, Spengler M, Jahn M, Niebuhr D, Haak T, Kulzer B, Hermanns N (2018). Adherence over time: the course of adherence to customized diabetic insoles as objectively assessed by a temperature sensor. J Diabetes Sci Technol.

[11] Keukenkamp R, Merkx MJ, Busch-Westbroek TE, Bus SA (2018). An explorative study on the efficacy and feasibility of the use of motivational interviewing to improve footwear adherence in persons with diabetes at high-risk of foot ulceration. J Am Podiatr Med Assoc.

[12] Rosenstock IM (1974). Historical origins of the health belief model. Health Educ Monogr.

[13] Chin YF, Huang TT, Hsu BR (2013). Impact of action cues, self-efficacy and perceived barriers on daily foot exam practice in type 2 diabetes mellitus patients with peripheral neuropathy. J Clin Nurs.

[14] Bann CM, Fehnel SE, Gagnon DD (2003). Development and validation of the diabetic foot ulcer scale-short form (DFS-SF). Pharmacoeconomics.

[15] Jarl GM, Heinemann AW, Norling Hermansson LM (2012). Validity evidence for a modified version of the orthotics and prosthetics users’ survey. Disabil Rehabil Assist Technol.

[16] Hagberg K, Brånemark R, Hägg O (2004). Questionnaire for persons with a Transfemoral amputation (Q-TFA): initial validity and reliability of a new outcome measure. J Rehabil Res Dev.

[17] Wallston KA, Stein MJ, Smith CA (1994). Form C of the MHLC scales: a condition-specific measure of locus of control. J Pers Assess.

[18] Samuelsson K, Wressle E (2008). User satisfaction with mobility assistive devices: an important element in the rehabilitation process. Disabil Rehabil.

[19] Ekback M, Benzein E, Lindberg M, Arestedt K (2013). The Swedish version of the multidimensional scale of perceived social support (MSPSS)--a psychometric evaluation study in women with hirsutism and nursing students. Health Qual Life Outcomes.

[20] Kroenke K, Spitzer RL, Williams JB (2001). The PHQ-9: validity of a brief depression severity measure. J Gen Intern Med.

[21] Vileikyte L, Gonzalez JS, Leventhal H, Peyrot MF, Rubin RR, Garrow A, Ulbrecht JS, Cavanagh PR, Boulton AJ (2006). Patient interpretation of neuropathy (PIN) questionnaire: an instrument for assessment of cognitive and emotional factors associated with foot self-care. Diabetes Care.

[22] Sullivan M, Karlsson J, Ware JE (1995). The Swedish SF-36 health survey--I. evaluation of data quality, scaling assumptions, reliability and construct validity across general populations in Sweden. Soc Sci Med.

[23] Coffey L, Mahon C, Gallagher P. Perceptions and experiences of diabetic foot ulceration and foot care in people with diabetes: a qualitative meta-synthesis. Int Wound J. 2019;16:183–210. 10.1111/iwj.13010 PMC7949356 30393976

[24] Chantelau E, Haage P (1994). An audit of cushioned diabetic footwear: relation to patient compliance. Diabet Med.

[25] Larsson J, Agardh CD, Apelqvist J, Stenstrom A. Long-term prognosis after healed amputation in patients with diabetes. Clin Orthop Relat Res. 1998:149–58. 9602814

[26] Monteiro-Soares M, Boyko E, Ribeiro J, Ribeiro I, Dinis-Ribeiro M (2012). Predictive factors for diabetic foot ulceration: a systematic review. Diabetes Metab Res Rev.

[27] Churchman N (2008). A retrospective audit of footwear use by high-risk individuals in north Derbyshire. Diabetic Foot J.

[28] van Netten JJ, Seng L, Lazzarini PA, Warnock J, Ploderer B (2019). Reasons for (non-) adherence to self-care in people with a diabetic foot ulcer. Wound Repair Regen.

[29] Paton J, Roberts A, Glasser S, Collings R, Marsden J (2014). “All I wanted was a pair of shoes”: a qualitative case study. Diabetic Foot J.

[30] Sabaté E (2003). Adherence to long-term therapies: evidence for action.

[31] van Netten JJ, Williams AE. Ensuring acceptance and wearing of pedorthic footwear with person-centred communication. In: Postema K, Schott K-H, Janisse D, Rommers GM, editors. Pedorthic footwear: Assessment and treatment. Foundation Berjalan; 2018.

[32] Jarl G (2018). Methodological considerations of investigating adherence to using offloading devices among people with diabetes. Patient Prefer Adherence.

[33] Jiang X, Wang J, Lu Y, Jiang H, Li M (2019). Self-efficacy-focused education in persons with diabetes: a systematic review and meta-analysis. Psychol Res Behav Manag.

[34] Mueller MJ, Smith KE, Commean PK, Robertson DD, Johnson JE (1999). Use of computed tomography and plantar pressure measurement for management of neuropathic ulcers in patients with diabetes. Phys Ther.

